# Field evaluation of seasonal trends in relative population sizes and dispersal pattern of *Aedes albopictus* males in support of the design of a sterile male release strategy

**DOI:** 10.1186/s13071-019-3329-7

**Published:** 2019-02-12

**Authors:** Gilbert Le Goff, David Damiens, Abdoul-Hamid Ruttee, Laurent Payet, Cyrille Lebon, Jean-Sébastien Dehecq, Louis-Clément Gouagna

**Affiliations:** 1 Institut de Recherche pour le Développement (IRD), UMR MIVEGEC (CNRS/IRD/UM): Maladies Infectieuses et Vecteurs: Ecologie, Génétique, Evolution et Contrôle, Montpellier, France; 2 IRD Réunion/GIP CYROI (Recherche Santé Bio-innovation), Sainte Clotilde, Reunion Island France; 3 Service de lutte anti vectorielle, Agence Régionale de Santé-Océan Indien (ARS-OI), Saint-Denis, Reunion Island France

**Keywords:** Mark-release-recapture, Male mosquito, Sterile insect technique (SIT)

## Abstract

**Background:**

To develop an efficient sterile insect technique (SIT) programme, the number of sterile males to release, along with the spatial and temporal pattern of their release, has to be determined. Such parameters could be estimated from a reliable estimation of the wild population density (and its temporal variation) in the area to treat. Here, a series of mark-release-recapture experiments using laboratory-reared and field-derived *Aedes albopictus* males were carried out in Duparc, a selected pilot site for the future application of SIT in the north of La Reunion Island.

**Methods:**

The dispersal, longevity of marked males and seasonal fluctuations in the population size of native mosquitoes were determined from the ratio of marked to unmarked males caught in mice-baited BG-Sentinel traps. The study was conducted during periods of declining population abundance (April), lowest abundance (September) and highest abundance (December).

**Results:**

According to data collected in the first 4 days post-release, the Lincoln index estimated population size as quite variable, ranging from 5817 in April, to 639 in September and 5915 in December. Calculations of daily survival probability to 4 days after release for field and laboratory males were 0.91 and 0.98 in April, respectively, and 0.88 and 0.84 in September, respectively. The mean distance travelled (MDT) of released field males were 46 m, 67 m and 37 m for December, April and September experiments, respectively. For released laboratory males, the MDT was 65 m and 42 m in April and September, respectively.

**Conclusions:**

Theoretically, the most efficient release programme should be started in July/August when the mosquito population size is the lowest (*c.*600 wild males/ha relative to 5000 wild males estimated for December and April), with a weekly release of 6000 males/ha. The limited dispersal of *Ae. albopictus* males highlights the nessecity for the widespread release of sterile males over multiple sites and in a field setting to avoid topographical barriers and anthropogenic features that may block the migration of the released sterile male mosquitoes.

**Electronic supplementary material:**

The online version of this article (10.1186/s13071-019-3329-7) contains supplementary material, which is available to authorized users.

## Background

The tiger mosquito, *Aedes albopictus*, is well adapted to domestic environments on La Reunion Island. It has a wide range throughout most of the coastal areas in the island and is abundant compared to other species. It causes considerable public health problems, reflected by its implication in the massive chikungunya epidemic occurring in the South West Indian Ocean (SWIO) islands from 2004 to 2007 [1], as well as being the primary vector associated with 231 reported indigenous cases of dengue fever in 2016 [2]. In addition to community education for source reduction, chemical control constitutes the most common method used for vector control by both larvicidal Bti (*Bacillus thuringiensis* var. *israelensis* toxins) and adulticidal treatment (deltamethrin) applied only in the vicinity of reported cases of arboviral disease [3]. However there are concerns over the non-specific nature of the chemicals used, including the potential selection for insecticide resistance that could affect their efficacy [4–6], in addition to the putative impact on human health and environemental ecosystems [7–9]. Moreover, controlling *Ae. albopictus* populations by breeding site elimination is difficult and time consuming due to their broad diversity of habitats and widespread distribution, sometimes in difficult-to-access locations. In recent years, research has been conducted to assess the feasibility of applying the sterile insect technique (SIT) in an integrated vector control approach targeting *Ae. albopictus* over wide areas in La Reunion. The SIT is a biological control method used to control insect pests by releasing a large number of sterile males into the wild population. These sterile males will compete with wild fertile males to mate with females in the field and thereby reduce the fertility of the target population [10, 11].

An adequate release strategy has to take into account the area to treat, the number and the quality of sterile males to release, the spatial (grid of releasing) and temporal (when to release, frequencies of release and duration of treatment) patterns of release [12]. The main purpose of this work was to realize a reliable estimation of the wild population density in the area to treat. Experiments were conducted by using the mark-release-recapture strategy (MRR), which constitutes a well established method for estimating the population size of a given species per unit area [13–20].

As well documented in previous studies, an *Ae. albopictus* population within a given habitat exhibits great variation in seasonal abundance in response to exposure to specific climatic conditions (e.g. temperature, relative humidity, rainfall and wind speed) and to several natural abiotic factors [21–23]. Therefore, for the purposes of the sterile insect technique, it is of utmost importance to know both the actual number of a given species per unit area, and trends in how population density changes in time and space. One way of obtaining the temporal trends of population abundance is by periodic release-recapture experiments at various times during the year. Information obtained is essential in determing the optimal moments for releases and in calculating the required rate of sterile insect release.

Here, a series of mark-release-recapture (MRR) experiments were carried out in different seasons with *Ae. albopictus* male samples from the field (F0), to estimate the seasonal change in population density. Unlike earlier studies [24, 25], the present study also focuses on a pre-release comparison of the survival and dispersal ability of laboratory reared and field derived *Ae. albopictus* males in a candidate urban field site chosen for the pilot testing of sterile male release. We hypothesized that the behaviour of colonized male mosquitoes may be modified by rearing processes under controlled conditions, which sometimes may result in a subsequent loss of natural traits through genetic selection [26–29]. More generally, comparison between laboratory-reared and wild mosquitoes in the field is important to assess any impact of raising inbred male populations on the ability to colonise and compete in natural settings.

## Methods

### Study area

The study was carried out in the site “Duparc”, a 22-hectare urban area located within the commune of Sainte Marie in the northern district of La Réunion. This site is one of the pilot sites chosen for field demonstration of *Ae*. *albopictus* suppression using sterile male releases. Within the study area *Ae. albopictus* is the sole *Stegomyia* mosquito species identified. Based on previous surveys (G. Le Goff, unpublished data), the peak of the population abundance usually occurs in summer between December and March, as measured by the degree of oviposition, followed by a sharp decline during the winter (between May and October) with the lowest level observed in August-September. In La Reunion, the climate is tropical with two main seasons: the austral summer between November and March-April is warm (with average temperatures of 26.0 °C, measured at the nearby Gillot aeroport) and very rainy; the austral winter, lasting from May-June to October, is cooler (with average temperatures around 22.5 °C) and drier [30].

### Production of experimental males

The study spanned from November 2015 to September 2016 with 3 mark-release-recapture (MRR) experiments carried out in different seasons with laboratory-reared and field-collected *Ae. albopictus* male mosquitoes*.* Field adult males were obtained from eggs collected by ovitraps within the study area during the 6 weeks before each experiment. When collected, eggs were dried for maturation under ambient condition for 7 days and then stored until the experiment. Around 40 ovitraps per week were placed in the field at different locations. Usually more than 50% of the ovitraps were positive. In addition, laboratory-reared eggs were collected from a colony established in 2010 from field-collected eggs. Both strains were reared as described previously [31], in a climate-controlled insectary (T: 27 ± 2 °C, RH: 75 ± 2%, 12L:12D light:darkness photoperiod). Random samples of both dried field-collected and colony eggs were hatched in tap water with dehydrated rabbit food (hay pellet, Compagnie des Grains du Capricorne, Le Port, Reunion Island). Upon hatching, larvae were reared to the pupal stage at a density of *c.*1000 larvae (L1) in 30 × 40 cm plastic trays each containing 2 l of water. They were fed with dry pellets composed of 50% rabbit-food and 50% fish-food (Sera Koi Food, Sera, Heinsberg, Germany). When pupae appeared, they were individually morphologically sexed under a stereomicroscope (Leica MZ6, Singapore, Singapore). Male pupae were allowed to emerge in plastic cages (30 × 30 × 30 cm, Bugdorm, MegaView, Science Education Services Co., Taichung, Taiwan) and emerging adults were provided continuous access to a 10% (w/v) sucrose solution soaked on wet cotton pads, which was placed for 2 days on the gauze top of the cage. The cages were checked for the presence of adult females (by observation of mating, blood plate attraction) and females were removed with an aspirator.

Two days prior to the release, all males of each group were marked with fluorescent dust (RADGLO® JST, Radiant N.V., Houthalen, Belgium). Two different colours of fluorescent dust were used to differentiate field males (yellow dye) from laboratory (red dye) ones. To achieve this, several batches of approximately 150 males each were aspirated from cages and put in a small paper pot covered with netting mesh. A 3-ml plastic pipette was used to transfer a cloud of fluorescent dust into the pot in a sufficient quantity to mark all males. The marking took place in the laboratory and batches of 1000–1300 marked males of each group (field and laboratory strains) were put in separate holding cages (60 × 60 × 60 cm). They were held under ambient laboratory conditions with access to a 10% sugar solution-soaked cotton pad for 24 h during which dead males of each group were removed and recorded.

### Field release procedure

The MRR procedure described below was repeated for three periods from November 2015 to September 2016 in order to assess the *Ae. albopictus* population density at different seasons. This timespan covered periods of population decline, lowest population and population increase. Specifically, MRR took place when the mosquito population was likely to be greatest, from 28th November to 4th December 2015 (beginning of rainy season), during the decrease in population size from 23th to 29th April 2016 (end of rainy season) and at the beginning of the summer when the densities are lowest, from 3rd to 9th September 2016 (during the austral winter). On each occasion, holding cages containing marked adult mosquitoes (usually two or three days post-emergence) were transported by car to the release site. The release of mosquito males occurred between 17:00 h and 18:00 h.

**Fig. 1:**
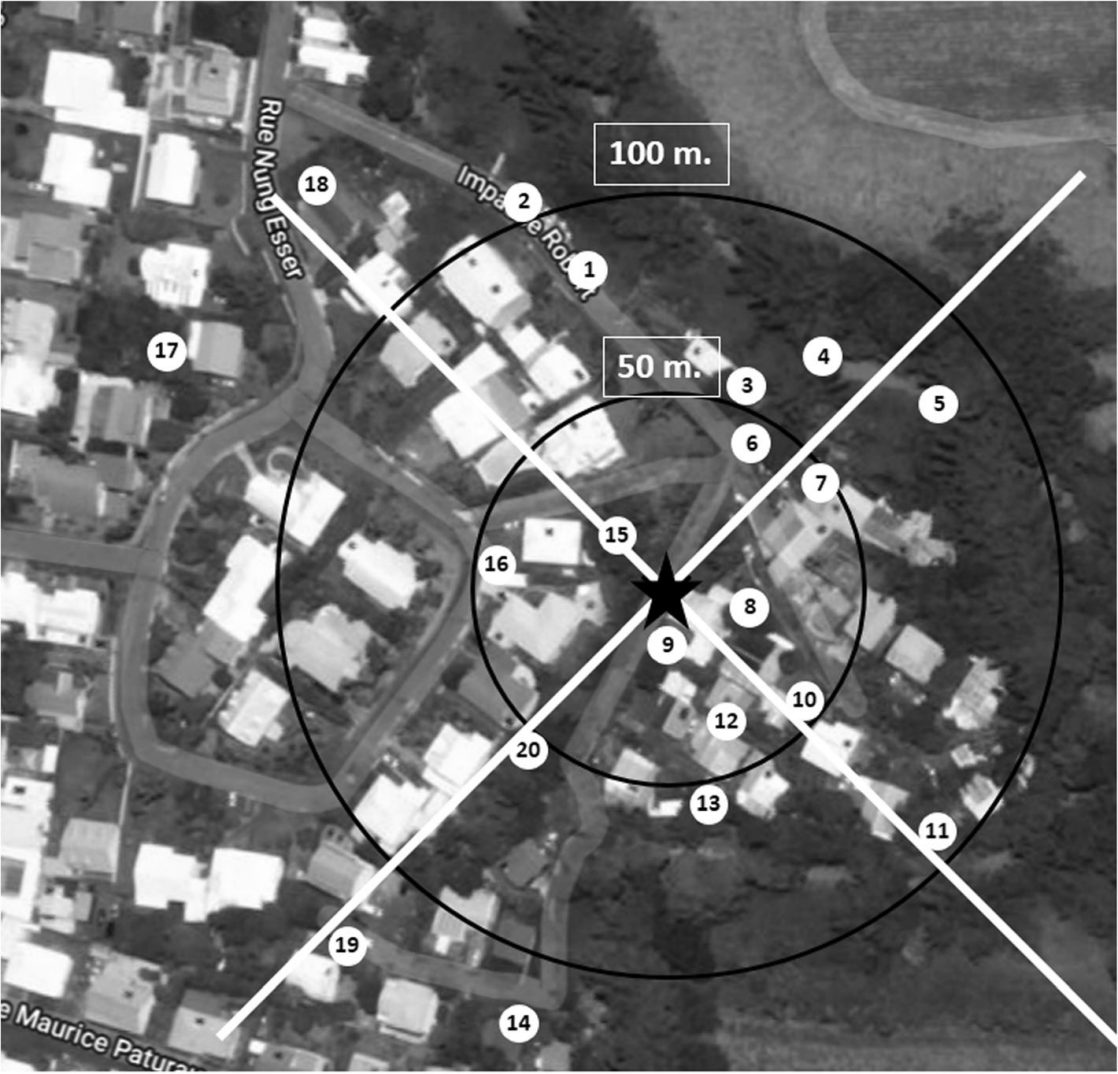
Geographical layout of the mark-release-recapture of field and laboratory-reared males of *Aedes albopictus* adults in the site of “Duparc”. The star indicates the position of the release site and the white circles indicate the recapture points with the number of traps. The circles represent the annuli (separated by 25 m) used to calculate the MDT (Google maps). The two white lines separated the region in four quarters representing the distribution of the traps according to the four directions

Figure 1 shows the geographical layout of the experiment. The release point marked in Fig. 1 (20°54'21.63"S, 55°31'26.5"E) was chosen to record the dispersion between house yards. During the three experiments, a total of 2493, 2731 and 1453 field males were released on 28th November 2015, 23th April and 3rd September 2016 (all Saturdays), respectively. For laboratory males, 2589 and 1497 males were released only on 23th April and 3rd September 2016, respectively. The release point and recapture positions were the same for the three experiments and locations were geo-referenced using a global positioning system (Garmin GPS 60).

### Recapture procedure

The recapture began approximately 40 h after each release. Mosquito collections were performed every day for four consecutive days using mice-baited traps. The classic BG-Sentinel trap (BG1) was modified to accommodate a cage containing three mice placed in a clear rearing polycarbonate cage as described in Le Goff et al. [32]. Live mice were used as the preferred attractant to ensure good capture results [32].

The mosquito sampling procedure consisted of a total of 20 mouse-baited BG sentinel traps that were deployed around the release point (Fig. 1). The traps were placed at ground level in a shaded location close to domestic areas. They were activated simultaneously every day between 9:00 and 10:00 h and mosquitoes were collected the day after at the same time. Batteries (12 V, 9 Ah) (FIAMM-AGM Technology, Aubergenville, France) were changed every day, while the group of mice used as bait were left in the field for the first two consecutive days and were replaced by new mice for the last two days. During daily trap inspection, mosquitoes found in the collecting bag were recovered, placed inside a plastic container, retrieved and brought to the laboratory for further processing. Field-collected mosquito samples were identified using morphological characteristics. Moreover, marked males were identified under stereomicroscopes using UV lamps (LF-104S, 245 nm, 4 W; Uvitec, Cambridge, UK). The total number of *Ae*. *albopictus* adults, the male ratio (defined as the number of males caught divided by the total number of *Ae*. *albopictus* adults caught) and the origin (field or laboratory) were recorded for each collection.

### Data analysis

Recapture rates were calculated as the proportion of the total number of marked males recaptured divided by the total number originally marked and released. As *Ae. albopictus* females were not released, we did not analyse data for captured females. Rather, the relative size of male populations was estimated only for the first day of recapture following each release and estimated for a 1 ha surface around the release point (a circle of 56 m diameter represents approximately 1 ha). The Lincoln index was modified for a low recapture rate [33] as

$$ \mathrm{P}=\left[{\mathrm{as}}^{\mathrm{t}}\ \left(\mathrm{n}-\mathrm{r}+\mathrm{l}\right)\right]/\left(\mathrm{r}+1\right) $$

where P is the estimated population density, a the number of marked males released, s the estimated probability of daily survival, t the sampling day post-release, n the total number of marked and unmarked males captured and r the number of marked males recaptured in BGS trap collections on the first day.

The daily survival probability (DSP) was calculated by regression of the total number of males transformed by log (x + 1) in all traps recaptured per sampling day. Survival probability was estimated from the result of the antilog of the slope of the regression line [34, 35]. The effect of male origin (field *vs* laboratory) on survival was analysed by comparing the slopes of the regression lines by the t-test. Average life expectancy was calculated by the formula: 1/-ln(DSP) [36].

For both daily survival and population size, dispersal distance was not taken into account, thus the number of mosquitoes recaptured at different distances were pooled.

Dispersal of the released males was calculated as the mean distance travelled (MDT) [33] that takes into account unequal trap densities within each annulus [37]. Here, concentric sampling annuli separated from each other by 25 m were established at 25, 50 and 75 m (as long as there was at least one trap in each annulus around the release points. Based on information provided in [33], annulus distances can be approximated as follows. MDT can be estimated according to the recaptures in those traps as follows (Fig. 1) [33]:

$$ \mathrm{MDT}=\left(\mathrm{Sum}\ \mathrm{for}\ \mathrm{all}\ \mathrm{annuli}\ \left(\mathrm{ER}\times \mathrm{median}\ \mathrm{distance}\ \mathrm{of}\ \mathrm{annulus}\right)\right)/\mathrm{Total}\ \mathrm{number}\ \mathrm{of}\ \mathrm{ER} $$

where ‘median distance of annulus’ is defined as: (distance inner radius + distance outer radius) / 2 and ER = ((number of mosquito recaptured in annulus)/number of traps in annulus) × CF. Annulus CF parameter corresponds to: (area of annulus/total trapping area) × total number of traps.

Besides the estimation of the dispersal of marked male mosquitoes, their directional movements within the study area were also examined and compared between laboratory- and field-derived males. For the direction of dispersal, proportions of marked mosquitoes caught in traps for the four days according to direction (North, East, South, West; see Fig. 1) were compared using the G-test [38]. We compared the observed counts of mosquitoes caught in each direction with the expected counts, which we calculated here as the theoretical expectation if the same proportion of mosquitoes was caught in each direction. As a *post-hoc* test, Chi-square tests were performed between proportions of mosquitoes in each treatment category.

## Results

### Recapture percentage

For all experiments, marked male mosquitoes were recaptured on every collection day following the release. Out of the 2493, 2731 and 1453 field *Ae. albopictus* males released during the three experiments, 148, 152 and 169 marked males (5.9%, 5.6% and 11.6%) were recaptured in the four days of collection in December, April and September, respectively. Out of the 2589 and 1497 laboratory males released during the second and third experiment, 107 and 178 marked males (4.1% and 11.9%) were recaptured in April and September, respectively (Additional file 1: Table S1).

Consistently in all experiments, the recapture rates following the release of marked males varied between days but remained similar in both wild and laboratory populations. The highest percentage of recaptures occurred on day 2 (between 31–40% of the total recapture) and the lowest on day 4 (between 8–22%).

### Male survival

**Fig. 2:**
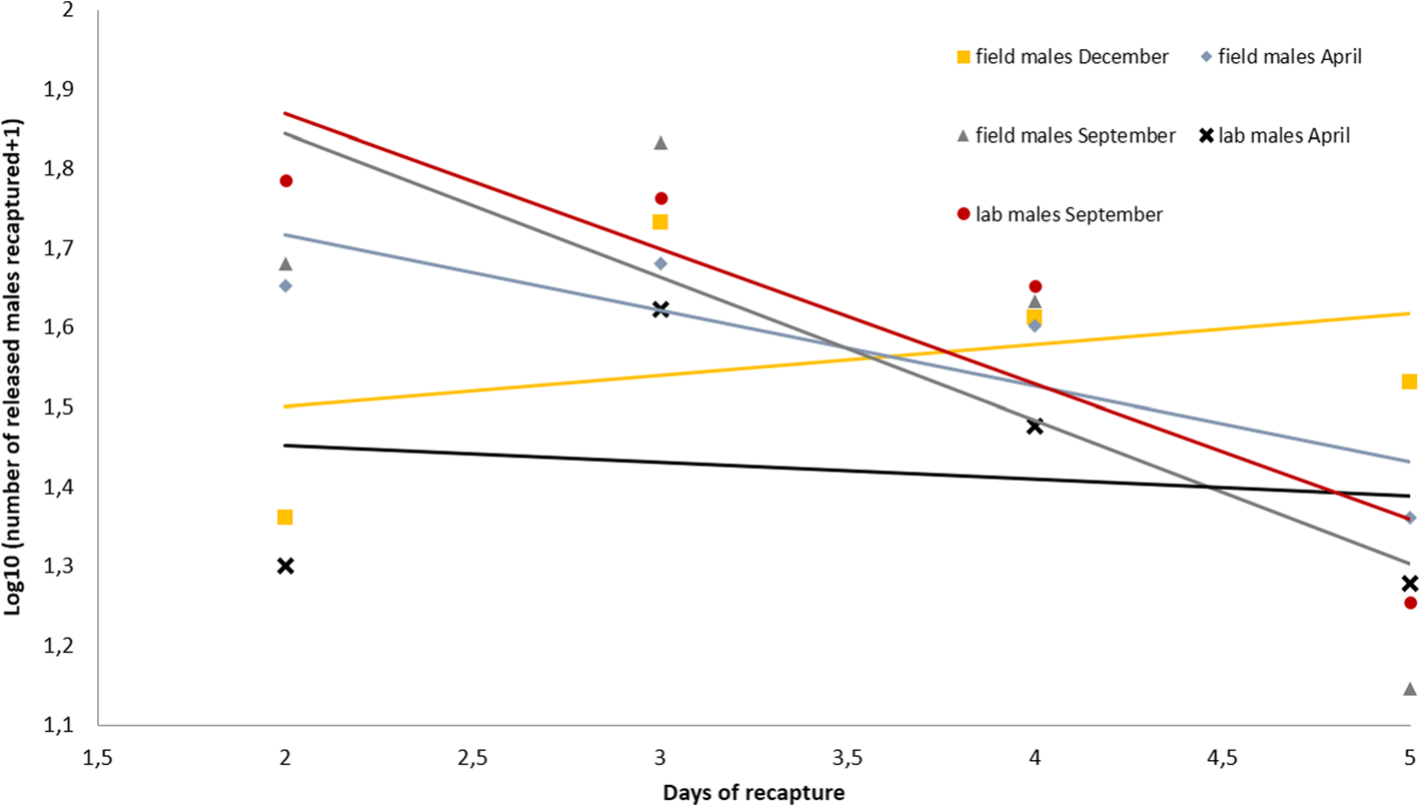
Regression lines of recaptures [expressed as log (number of released males recaptured + 1)] of cohorts of field-collected and laboratory-produced *Aedes albopictus* released in the three experiments. The equations of regression lines are: for field males December: y = 0.039x + 1.4232, for field males April: y = -0.0954x + 1.9083, for laboratory males April: y = -0.1804x + 2.2049, for field males September: y = -0.0213x + 1.4946 and for laboratory males September y = -0.17x + 2.2094. The antilog of the slopes of regressions lines gives the daily survival probability

Figure 2 shows the survival probability of field and laboratory males in each treatment category based on the regression models. For the first experiment, because of the relatively low recapture rate on the first day, the slope of the regression line for adult recaptures by day was 0.039 and yielded the impossible daily survival probability of 1.04. If the first day was removed, the calculated survival probability was 0.90 giving an average life expectancy of 9.5 days. For the second experiment, in April 2016, a daily survival probability of 0.91 (life expectancy 10.6 days) and 0.98 (life expectancy 49.5 days) were calculated for field and laboratory males, respectively. For the last experiment, in September 2016, a daily survival probability of 0.88 (life expectancy 7.8 days) and 0.84 (life expectancy 5.7 days) were estimated for field and laboratory males, respectively.

### Population estimation

Using the Lincoln index, the population estimates calculated on the first day of capture were evaluated at 5915 and 5817 males/ha at the beginning and the end of rainy season in December 2015 and April 2016, respectively. At the end of dry season, in September 2016, the population size was estimated at 639 males/ha. Variation in the Lincoln index was observed according to the recapture day: the estimation of male populations varied from 5900 to 28000, from 5800 to 24000 and from 600 to 11000 for December, April and September, respectively.

### Dispersal

**Fig. 3:**
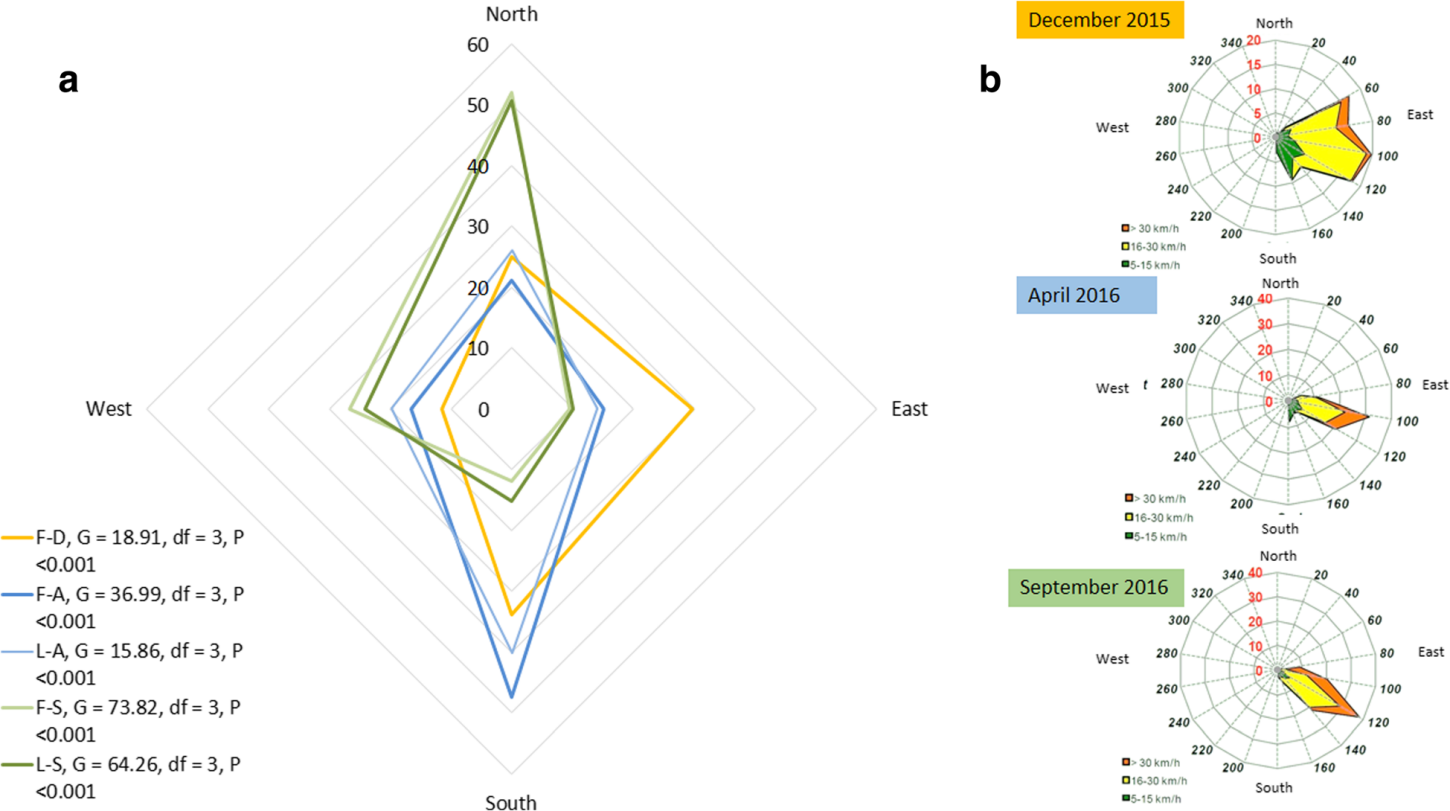
**a** Proportions of marked mosquitoes caught in traps for the four days according to the direction for the different treatment: F-D, field males December; F-A, field males April; L-A, laboratory males April; F-S, field males September; L-S, laboratory males September. The G-test was used to determine if the same proportion of males dispersed in all directions (*P* < 0.001 indicates a strong significant difference with a ratio of 1:1:1:1 in all directions). **b** Wind roses for the three dates of MRR. Frequencies of direction and speed of the wind during one month, with mean values calculated every 3 h [30]

All studied *Ae. albopictus* males dispersed in all directions but not homogeneously (see G-test in Fig. 3a). Males also dispersed differentially according to the season in which they were released. However, within each season, field and laboratory males released dispersed similarly (G-test, *G* = 4.43, *df* = 3, *P* = 0.22 and *G* = 1.19, *df* = 3, *P* = 0.59 for April and September, respectively). The directions of the prevailing winds at Gillot airport (at 1000 m from the release site) are presented in Fig. 3b for each MRR period. For the three experiments, the prevailing winds were in the direction east-southeast.

During the austral summer (December), 88% of the marked field males were collected in the first 50 m around the release point and the number decreased for the following distances (Table 1). At the end of the austral summer (April), most of the marked field and laboratory males were found in the first 25, 50 and 75 m around the release point. During the austral winter season (September), more than 90% of the field and laboratory males were found in the first 50 m around the release point.

**Table 1:** Number of marked males collected in traps according to the distance from the release point

Month	Type of male	Distance of collection					
		25	50	75	100	125	150
December	Field	41	48	7	3	1	0
April	Field	31	33	28	3	2	3
	Lab	18	41	35	0	4	2
September	Field	34	59	5	1	1	0
	Lab	29	61	4	5	1	0

As an example, Table 2 shows the step by step calculation of the mean distance travelled (MDT) for released field males in April 2016 based on the recapture rates, accounting for the distance from the release point. The MDT of released field males was 46 m, 67 m and 37 m for the December, April and September experiments, respectively. For released laboratory males, the MDT was 65 m and 42 m for the April and September experiments, respectively. The MDT of field and laboratory males were similar at the end of the warm and rainy season (April) and at the end of austral winter season (September).

**Table 2:** Step-by-step calculation of the mean distance travelled (MDT) of field *Aedes albopictus* males released in the first experiment (April 2016)

	Annulus						
	1	2	3	4	5	6	Total
A. Radius inner (km)	0	0.025	0.05	0.075	0.1	0.125	
B. Radius outer (km)	0.025	0.05	0.075	0.1	0.125	0.150	
C. Area (km^2^)	0.001964	0.005891	0.009818	0.013745	0.017672	0.0251599	
D. Area total (km^2^)							0.0707
E. Number of traps	3	4	5	3	3	2	
F. Total number of traps							20
G. CF = (C/D)*F	0.55	1.67	2.78	3.89	5.00	6.11	
H	47	51	43	4	3	4	
I. ER = (H/E) * G	8.70	21.25	23.89	5.19	5.00	12.22	
J							56.20
K. Distance (A+B)/2	0.0125	0.0375	0.0625	0.0875	0.1225	0.1375	
L	0.109	0.799	1.493	0.454	0.562	1.680	
M							5.095486
MDT							0.066826

Letters represent: A, inner radius of each annulus; B, outer radius of each annulus; C, area of each annulus; D, area total of the annuli; E, number of recapture sites in each annulus; F, the total number of traps; G, correction factor for each annulus; H, number of *Ae. albopictus* marked males recaptured in each annulus during the four days of the experiment; I, ER estimated recaptures as a total recapture; J, annuli sums totalled; K, median distance of each annulus; M, median

## Discussion

The results obtained from a series of mark-release-recapture (MRR) experiments with *Aedes albopictus* male mosquitoes in different seasons, showed seasonal changes in population size in the studied urban settlement. The evaluation of population sizes in terms of *Ae. albopictus* male numbers per unit area shows high densities in summer reaching 6000 males per hectare, while densities were ten-fold lower in the winter.

### Recapture rates

For released males derived from the field population, recaptures rates were equivalent in December and April (5.9 and 5.6%, respectively) and increased in September (11.6%). For laboratory reared males, the recapture rate was also higher in September than in April 2016. Seasonal conditions could explain the evolution of the recapture rates between MRR experiments. The dry conditions in September could have led mosquitoes to fly for less time, and search for resting sites quickly after release. The males could have stayed near the release point and be caught proportionally more often than during the other periods. Direct comparisons with recapture rates obtained in previous MRR experiments for *Ae*. *albopictus* should be employed with caution due to high levels of variability between experimental parameters (number of traps, types of traps, frequency of captures, etc.) and experimental conditions (e.g. landscape, ecological and climatic conditions). This is also true for comparisons between dispersal ability, survival and population estimation. Recapture rates of males observed here were comparable to those obtained in La Reunion Island by similar mouse-baited BG-sentinel traps (6.4–15.8% [24]) or in an MRR study in Missouri, USA where *Ae*. *albopictus* was recaptured by vacuum aspiration at a scrap tyre yard and in forest vegetation (4.1% in 1989 and 10.1% in 1990 [36]). It is worth noting that the presented results are much higher than those obtained using sticky traps in Italy (1.1% [39]).

### Population size estimates

Knowledge of *Ae*. *albopictus* population size and its seasonal fluctuation at the site of Duparc is needed to determine the starting date for a SIT programme and the intensity of sterile malesʼ release. Expressing population size estimates as population densities could exhibit some bias as the site is not a closed area with distinct ecotonal borders; nonetheless, this estimation provides a reasonable and robust indication of population density fluctuation according to season. In brief, the population estimation for one hectare is around 6000 males for the experiment in December (summer, beginning of warm and rainy period) and April (at the end of summer) while ten times less in September (at the end of winter, the dry and cooler season). During the previous two years, a trend for a decrease between December and April was observed with three times less mosquitoes in April compared to December (G. Le Goff et al., unpublished data). The absence of differences between December and April in our experiment could be explained by the homogeneous weather conditions during these months. More specifically, during the two to three weeks preceding the entomological surveys, rainfall was scant (rainfall 20.4 and 29.7 mm in November 2015 and April 2016, respectively) and the average temperatures were comparable (25.2 and 26.0 °C in November 2015 and April 2016, respectively). In addition, there was relatively weak rain in summer 2015–2016 (rainfall < 1 m, between October 2015 and April 2016) with few rains and a total absence of cyclones or summer storms. This situation applied for the whole of the beginning of 2016. The winter was normally dry leading to a drastic reduction in the number of breeding sites [40] and probably to flying insects having shorter lifespans. Consequently, this may have led to a decrease in populations of *Ae*. *albopictus* adults and larvae. A previous MRR experiment [24], performed at a peri-urban non-residential area in the south of La Reunion Island, also showed a marked difference in *Ae. albopictus* population densities between dry and wet seasons.

### Survival

Released males were recaptured until six days after release, suggesting that they were able to find sugar sources, essential for survival and sexual maturation [41]. Indeed, in a laboratory setting where males have access to no sugar but only water, all males died within three days [42]. The daily survival index, around 0.9, and the calculated life expectancy estimated from the MRR confirm these observations. The probability of daily survival was similar for field and laboratory males of *Ae*. *albopictus*, suggesting that colonization and laboratory rearing did not alter survival. Moreover, survival in the dry season (September) appeared to be lower than during the wet season, as observed by Lacroix et al. [25]. In this study, the life expectancy of males was less than one week during the dry season and around three weeks during the wet seasons in a residential area of Saint-Pierre, in the south of the island of Reunion [25]. However, usually, daily survival is calculated from the slope of the regression line for adult recaptures over ten to 21 days to be able to estimate the disappearance of the marked males over the time. In our experimental conditions, insects were collected only for four days. Whilst this duration does not allow a good estimation of life expectancy, it gives a good idea of the survival of the males just after release, an essential period for the efficiency of the SIT method. In future experiments, to estimate the longevity of irradiated males, a longer period of recapture will be used.

### Dispersal and mean distance travelled

In all experiments, marked males from the field and from the laboratory dispersed similarly in all cardinal directions relative to their release site, suggesting good mixing with the wild populations. However, males dispersed differently according to the season, probably due to the different meteorological conditions. However, this difference in dispersal is not explained by the direction of the prevailing winds or the force of the winds (recorded in Gillot airport 1000 m from the release site) except in April 2016 when the direction of the prevailing winds at Gillot airport was northwest [30] (Fig. 3). We can hypothesize that the prevailing winds recorded at Gillot in an open area could have been modified by the urban landscape. Another hypothesis is that directional biases in dispersal could depend more on the terrain features and habitat selection by released lab-reared and wild mosquitoes.

The mean distance travelled estimated for both field and laboratory males were similar for both experiments in April and September (around 65 m and 40 m, respectively). These results suggest that rearing process does not influence the flying ability of laboratory produced males. Our observations are similar to those found in vegetated area by Lacroix et al. [25] who obtained a value of MDT for *Ae*. *albopictus* ranging between 29–46 m in the south of La Réunion or Takagi et al. [43] who obtained less than 36 m in a grassy and scrub area. However, when compared with results from MRR in urban sites, our MDT is lower than the 97–212 m found by Bellini et al. [44]. Strong differences in the structure of urban areas could explain these differences. Indeed, the shape and position of buildings, position of squares, gardens and main roads could create diverse topographical barriers [44].

Evidence of the limited dispersal of *Ae. albopictus* males up to four days post-release suggests a SIT programme would need a widespread release of males over the field area. It is indeed important to take into account the natural barriers and anthropogenic features that may block mosquito movement. Further MRR experiments with the release of sterile *Ae. albopictus* males are planned in order to optimize our strategy, notably the grid of release.

## Conclusions

An important utility of the results is to rationalize the release scenario by collecting, analyzing and interpreting relevant field data and by developing a release strategy that can help to effectively control *Ae. albopictus* population using SIT in Duparc, a candidate field selected for SIT testing in the North of La Reunion Island. In this site, the estimate of *Ae. albopictus* population density obtained from mark-release-recapture data indicated that a less costly and more broad and effective applicable release programme should start in July-August when the mosquito population size (*c.*650 wild males/ha) is the lowest (relative to 6000 wild males estimated for December and April), and must be continued for several months to avoid the sudden increase in population abundance with the advent of the warm and rainy season. However, the duration of the release programme could not be estimated from the present results. Modelling and field trials are warranted to achieve a better understanding for the duration of the release programme required. The distance between sterile male release points and the timing of the releases are two crucial factors in planning SIT programmes [44]. The number of males that should be released depends on population density, sexual competitiveness of males and the percentage of females with which these males can mate. Damiens et al. [26] showed that in laboratory conditions, a ratio of 10:1 (sterile male: wild male) induced sterility in 62 ± 11 % of the female population. Assuming this level of sterility is enough to significantly reduce a wild population (if not, the ratio should be higher), this suggests that at least 6000 sterile males per hectare (i.e. ten times more sterile males than the wild males) per week would be required to achieve meaningful population suppression in the urban pilot site of Duparc. With a surface of 22 ha, a minimum of 132,000 males per week will be needed for release in the whole site. This estimation will not be applicable without further information on the longevity and dispersal of released sterile males *Ae. albopictus* gained from periodic release-recapture tests. This will be considered as an important prerequisite for designing a mass-rearing system at an appropriate scale and subsequent larger-scale SIT interventions for vector control.

## Additional file

Additional file 1: **Table S1.** Total data set of released males *Aedes albopictus* recaptures according to the distance of the traps from the release point and the date of experiment. (XLS 53 kb)

## Abbreviations

BG: Biogent
BGS: Biogent-sentinel trap
FIAMM-AGM: ‘Fabbrica Italiana Accumulatori Motocarri Montecchio’-absorbent glass material technology
MDT: Mean distance travelled
MRR: Mark-release-recapture
SIT: Sterile insect technique
